# Behcet's Disease: Is There Geographical Variation? A Review Far from the Silk Road

**DOI:** 10.1155/2015/945262

**Published:** 2015-12-20

**Authors:** Nieves Marie Leonardo, Julian McNeil

**Affiliations:** ^1^Department of Rheumatology, Modbury Hospital, Smart Road, Modbury, SA 5092, Australia; ^2^Discipline of Medicine, Faculty of Health Sciences, University of Adelaide, Modbury Hospital, Modbury, SA, Australia

## Abstract

Behcet's Disease (BD) is a systemic vasculitis characterized by the triad of recurrent mouth and genital ulcers with eye involvement. To date there are no laboratory tests specific for the disease and diagnosis continues to remain on clinical grounds. Multiple criteria have been created as guides for diagnosis; however, given the wide spectrum of organ involvement, some cases remain undiagnosed. The diagnosis of Behcet's Disease may only be made over time as the clinical manifestations emerge sometimes separated by months and even years. With an increased recognition of this disease it has become apparent that there is geographical variation in clinical manifestations. In particular cardiac manifestations are not seen commonly in Caucasians compared to Asian and Middle Eastern patients, while neurological manifestations are more common in Caucasians. Use of immunosuppressive and immunomodulatory drugs to suppress inflammation remains the cornerstone of treatment.

## 1. Introduction

Behcet's Disease (BD) is a chronic, multisystem vasculitis. It is categorized under variable vessel vasculitis in the new Chapel Hill nomenclature as it involves blood vessels of any type and size [1]. It is characterized by relapsing aphthous ulcers commonly occurring in the oral mucosa and genitalia with ocular involvement. Other organ systems may be involved anytime throughout the course of the disease [2].

The disease was named after Hulusi Behcet, a Turkish dermatologist who in 1937 presented three cases of patients he followed for years with a triad of oral ulceration, genital lesions, and recurrent eye inflammation [3]. He thus became the first physician who brought it to the attention of the medical community. However, in 1930, a Greek ophthalmologist named Benediktos Adamantiades described a case of relapsing ocular symptoms with associated genital ulceration and arthritis in a 20-year-old male patient [4]. For this reason it is also known as Adamantiades-Behcet's Disease.

## 2. Epidemiology

Behcet's Disease has a worldwide distribution (see Table 1). However, it is observed commonly among populations living along the historic Silk Road, an ancient trading route that spans from Japan and China in the Far East to the Mediterranean Sea, including countries such as Turkey and Iran. That is also why it was given the name “Silk Road Disease” [5].

It is most common in Turkey with estimated prevalence of 421 per 100,000 population followed by Iran, Israel, and Japan [6]. It remains rare in other countries such as the Americas and African countries [5] as well as in Australia for which the prevalence remains unknown but with an annual incidence of 0.6% [7]. A higher percentage of cases in low-prevalence countries (see Table 1) are seen in patients whose ancestry is traceable to high-prevalence areas [8].

The mean age of onset is from the 2nd to 4th decade of life. Behcet's Disease rarely occurs in children and in patients over 55 years old [3, 9]. There is a male preponderance in the Mediterranean region and women are more commonly affected in the Far East [10]. It has a chronic course with unpredictable exacerbations and remissions.

## 3. Aetiology

The exact cause is unknown. However, combination of genetic and environmental factors is likely to play a role. Some researchers believe that an environmental factor or infection, such as a virus, bacterium, or pollution, may trigger an autoinflammatory reaction within people who have certain disease susceptibility genes [2]. It primarily affects vascular endothelial cells leading to inflammation and thrombosis [11]. Serum levels of neutrophil priming cytokines such as TNF, interleukin B, and IL-8 are known to be raised in patients with Behcet's Disease and significant neutrophil infiltration is found in early lesions [12].

Several genes have been found to be associated with the disease, the most common being in the HLA-B class. The most strongly associated known gene associated with Behcet's Disease is HLA-B51 [3, 13]. It functions as an immunogenetic marker for a small group of patients along the Silk Road [14]; however, this association is not seen in Caucasian patients [15] and accounts for less than 5% in familial cases [3].

## 4. Diagnosis

To date, Behcet's Disease remains a clinical diagnosis based on its disease manifestations. There is no relevant biological test for diagnosis. For this reason different classification criteria were created for the identification of Behcet's Disease for the past nine decades (see Table 2).

All criteria have in common the fact that they give significant weight to recurrent oral ulceration. However, recurrent oral ulceration is not an uncommon complaint and differentiating it from the recurrent oral ulceration of Behcet's Disease can be a challenge. In 1990 a group of scientists came together to form the International Study Group (ISG) for Behcet's Disease [16]. The ISG defined a set of international classification criteria which is designed for research studies, which at times is used in clinical settings as a basis for diagnosis of Behcet's Disease. The features were defined as the presence of particular symptoms as seen in Table 3.

A total ≥3 is said to be diagnostic. However, it did not allow for variation in the symptoms of the disease, since an oral ulcer was required for the diagnosis [17].

The appropriateness of the ISG criteria as a diagnostic tool has been questioned [18], and to accommodate for the geographical variation as well as global distribution of Behcet's Disease, an international team from 27 countries was formed to reassess existing criteria. The major criteria (oral aphthosis, genital aphthosis, and ocular lesions) were each given 2 points, whereas 1 point was assigned to the other manifestations. Due to geographical variation, the pathergy test was given 1 point [17].

In the new International Criteria for Behcet's Disease (ICBD) (see Table 4), a pathergy test was optional, as this takes into account the declining sensitivity and increasing specificity of pathergy test [19]. The reported higher sensitivity of the ICBD will allow for earlier recognition, earlier diagnosis, and earlier treatment [17]. However, the fact that major manifestations of Behcet's Disease can emerge at different points in time in the course of the disease needs to be considered. While eye lesions typically occur after the onset of oral ulceration, the delay between the two manifestations may take a decade [20].

Inflammatory markers, mainly erythrocyte sedimentation rate (ESR) and C-reactive protein (CRP), have long been recognized as inaccurate indicators of disease activity. However, there was a consensus that prompt investigation is needed if there is a significant elevation from baseline [21].

## 5. Clinical Manifestations

Behcet's Disease is a vasculitis that affects both arteries and veins of all sizes thus causing a diverse spectrum of organ involvement from head to foot that can emerge at any point in time. These systemic manifestations lack diagnostic reliability [2] and are primarily established based on clinical grounds in the absence of an alternative explanation [16].

### 5.1. Mucocutaneous Manifestations

Mucocutaneous lesions are the established hallmark for the diagnosis of BD [3]. Oral ulceration is the most constant symptom, seen in over 97% of patients [9]. It is a painful, recurrent, round or oval ulceration with well-defined borders and central yellowish pseudomembrane. In each attack, ulcers may vary in number and size from 1 mm to 30 mm in diameter. They heal spontaneously within 3 weeks with no scarring. It is difficult to differentiate them from ulcers due to other causes but their characteristics and recurrence should prompt clinicians to look for other systemic signs of BD [3].

Ulcers similar to those seen in the mouth occur on the vulva or vagina in the female, and on the scrotum or penis in male, but may leave a scar. Genital ulcers can be seen in 60% to 89% [9] of cases and are suggestive of the diagnosis of Behcet's Disease [3]. Genital ulceration is more commonly seen in western countries and can occur close to the anal sphincter [9].

### 5.2. Skin Manifestations

The most frequent skin manifestations are pseudofolliculitis and erythema nodosum. Pseudo­folliculitis (pustulosis) is characterized by a dome shaped sterile pustule on a round erythematous-edematous base that is indistinguishable from acne vulgaris, whilst erythema nodosum is characterized by painful multiple subcutaneous nodules that vary in size and change in colour as they age [9]. They can appear all over the body [3] and are frequently seen at the lower extremities.

The pathergy reaction is included in the criteria for the diagnosis of Behcet's Disease as well as an indicator of its activity [18]. It is a nonspecific hyperreactivity of the skin to minor trauma such as a needle prick. A papule or pustule containing sterile pus typically forms 24 to 48 hours after an intradermal injection of the skin with a 20-gauge needle [22]. Although more than 50% of patients from the Silk Road (Turkey and Japan) have a positive pathergy test, it is rarely observed in patients from Northern Europe, USA [23], and Australia [18]. It used to be an important diagnostic criteria for Behcet's Disease; however, the frequency of the pathergy phenomenon was reported to decrease during the past decades [19].

### 5.3. Ocular Manifestations

Uveitis occurs in approximately two-thirds of patients with Behcet's Disease. It is usually bilateral but is seldom the initial manifestation. It can occur decades after the initial manifestation of oral ulcers [20]. A single attack will usually heal spontaneously without producing any sequel [9]. However, the characteristic ocular feature is a relapsing uveitis that may involve the anterior segment, posterior segment, or both [20], including occlusive retinal vasculitis [24] which can lead to blindness if treatment is delayed.

Uveitis is an inflammatory lesion producing pain, photophobia, and visual disturbances [9]. It appears like dust particles in a sunbeam that are best seen with a narrow slit-lamp. It can be associated with a layer of pus in the anterior chambers (hypopyon). With intensive therapy and careful care the prognosis is considerably improved with only 2% developing blindness at 6 years of follow-up [3].

### 5.4. Cardiac Involvement

Cardiac involvement is uncommon. It was initially described by Mirone et al. [25] in 1958 as a case of paroxysmal fibrillation and heart block in a patient with Behcet's Disease. This was followed by a case of myocardial infarction complicated by incomplete right bundle-branch block in 1963 by Oshima et al. [25]. Since then different types of cardiovascular lesions, including pericarditis, atrial thrombus, complex ventricular arrhythmia, myocardial infarction, heart block, and sudden death, have been reported in association with Behcet's Disease [26]. Previous studies suggested that QT dispersion and corrected QTc dispersion parameters are significantly greater in patients with Behcet's Disease than control subjects [27, 28]. Others believe that cardiac arrhythmias such as supraventricular tachycardia may signify an underlying active inflammation which may represent the anatomic basis for a reentrant atrial circuit or enhanced automaticity giving rise to arrhythmia [25]. Eryol et al. [27] concluded that proximal atrioventricular complete heart block may develop without other signs of cardiac involvement, possibly from inflammation of the conduction system.

### 5.5. Articular Manifestation

Articular disease is common, occurring in almost half of patients. It can be the presenting feature, long before the other manifestations [3]. Most patients suffer from a nonerosive, nondeforming oligoarthritis typically involving the knees, ankles, and wrists [30]. Rarely, it can present as sacroiliitis [31] or erosive arthritis [29]. Myopathy has been reported [32].

### 5.6. Neurologic Manifestations

Neurologic manifestations can occur within 10 years of disease onset. They are observed in 20% to 40% of cases with a male preponderance [33]. The most common neurological features are headache, cranial nerve signs, ataxia, and sensory deficits. Parenchymal disease is more common and hemisphere lesions manifest particularly as stroke-like syndromes and seizures [34].

Prognosis is generally poor but with prompt and intensive immunosuppressive therapy improvement can be observed [3].

### 5.7. Gastrointestinal Involvement

Gastrointestinal manifestations can be seen in 7–29% of patients. They are produced by ulcers anywhere in the gastrointestinal tract. The classical form is ulceration of the ileocecal region. Patients can present with abdominal pain, diarrhea or constipation, or even acute abdomen due to perforation at the site of an ulcer [35]. It is difficult to differentiate Behcet's Disease from inflammatory bowel disease because of the similarity in intestinal and extraintestinal symptoms. Nevertheless, presence of granulomata can be used to exclude Behcet's Disease [3].

### 5.8. Vascular Involvement

Vasculitis is the pathognomonic finding in BD. Deep vein thrombosis is the main feature [9]. Although venous involvement is more common, it can affect both the veins and arteries and capillaries [3]. Venous thrombosis may occur at any site and may even involve large vessels such as the inferior vena cava, the superior vena cava, and the pulmonary artery. Thrombophlebitis frequently relapses and occurs generally in the first year after onset [3].

Arterial involvement is seen in 3 to 5% of cases [36]. Aneurysm formation accounts for most manifestation. Patients can be asymptomatic [37] and they can rupture suddenly. Vascular surgery is mandatory but relapse at the site of bypass is frequent [3].

### 5.9. Pulmonary Manifestations

Pulmonary manifestations are rare and dominated by vascular involvement. Hemoptysis is the main manifestation and can be massive and fatal [37].

### 5.10. Genitourinary Manifestations

Genitourinary involvement, other than aphthous ulceration, is rare. It can occur as recurrent epididymitis or nephropathy [3].

## 6. Geographical Variation

Disease presentation and manifestations vary in different ethnic groups and in different countries [38]. Although recurrent mouth ulcers were found almost universally, other manifestations are more common in the Far East. In Japan, Behcet's Disease is one of the three most frequently diagnosed causes of uveitis in patients [20], while it is rare in Australia [7]. No case of Behcet's Disease as a cause of uveitis has been found in an indigenous Australian [39].

The pathergy reaction is considered highly sensitive and specific for Behcet's Disease in patients originating from the Silk Road but is often negative from patients from the West. The frequency of HLA-B51 also varies by region. Greek patients have a significantly higher prevalence of HLA-B51, but the prevalence in the patients in the UK was significantly lower [20]. A few studies have demonstrated a male preponderance of patients with BD in Middle Eastern countries while a female preponderance is generally seen in Asian countries [2].

In the retrospective study done by Joseph and Scolding [34] it was suggested that there are geographical variations in the neurological manifestations of Behcet's Disease. Although there are no significant differences regarding neurological features of Behcet's Disease between Caucasians and those of Middle Eastern origin, their study concluded that presentation of neurological features was more common in Caucasians than Middle Eastern patients. Frequency of seizures was found to be sevenfold higher than in Turkish series [34].

Genital ulcers are more commonly seen in Caucasians [9]. Caucasians have a 14-fold relative risk of developing venous occlusion and a 5.4-fold relative risk of suffering from an arterial event [40].

BD has a global distribution but its prevalence is geographically variable (see Table 1) [13]. Despite the differences in prevalence among different ethnic groups, a number of previous studies have reported no or only few variations in the clinical characteristics of BD in different regions [15, 33].

Lewis et al. in 2007 [38] reached a different conclusion, as they found no differences in systemic manifestations comparing patients of different ethnic origin in the same country or in case series from different countries. They reported that evidence is lacking to support the concept that there are different expressions in the clinical phenotype of BD in different ethnic groups [38]. They concluded that differences between ethnic groups arise with respect to HLA-B51 and pathergy tests but not systemic manifestations.

## 7. Treatment

Treatment is usually focused on symptomatic management, improving quality of life, and preventing irreversible damage. It is mainly based on the suppression of the inflammatory attack using immunomodulatory and immunosuppressive agents such as corticosteroids, azathioprine, or interferon *α*. An example of this is that it is not suggested to use antiplatelet, anticoagulant, or antifibrinolytic in the case of venous thrombosis as the main pathology in Behcet's Disease is inflammation of the vessel wall [41]. At the same time, the management approach typically involves teamwork from different medical specialties such as ophthalmology, dermatology, and rheumatology with input from neurology, cardiology, and gastroenterology as required [42].

In 2008 the European League Against Rheumatism (EULAR) formed a committee to develop evidence-based recommendations for the management of Behcet's Disease [41]. Recommendations related to the eye, skin, mucosal disease, and arthritis are primarily evidence-based, but recommendations on vascular disease and neurological and gastrointestinal involvement are based mainly on observational studies, retrospective analyses, and expert opinion [41].

The first-line treatment for mucocutaneous manifestation of Behcet's Disease is colchicine (1 mg/day). For joint manifestations NSAIDs are usually sufficient [9]. It should be noted that although cyclosporine A is used for vascular involvement and refractory eye disease, it should not be the first-line treatment for any patient with neurological involvement due to the possibility of neurotoxicity [41]. In all cases, if the disease is resistant or becomes resistant, to these treatments, biologic agents are the last resort [42] (see Table 5).

Barry et al. [42] have suggested that the guidelines for the management of BD need to be updated. Currently the EULAR recommendations constitute “the gold standard” [42]. However, clinicians should be aware of the limitation of the existing guidelines as the last literature review regarding management was done a decade ago and new studies were published since then. Updated treatment, recommendation, and management guidelines are needed.

## 8. Prognosis

Panuveitis and retinal vascular occlusion were significantly more frequent in men than women, and men tended to have a worse visual prognosis [20]. Whilst mucocutaneous disease is indeed the most common manifestation of BD, it is the cardiovascular and neurological disease which has the potential to cause most morbidity and mortality [43].

## 9. Discussion

Australia is a country populated by migrants some of whom originate from countries along the Silk Road. Despite that, Behcet's Disease is reported to be rare in Australia. However, recurrent mucocutaneous ulceration, uveitis, and arthritis are a common presenting complaints. In low-prevalence countries such as Australia the challenge for the clinician is to be able to recognise the pattern of the disease as the presenting manifestation could be different from that of the Silk Road. This was highlighted in the study done in Brazil [44], where a study was conducted in a stomatitis centre for a year; 50 patients were identified with recurrent aphthous ulcer (RAU) but only 1 patient (2%) fulfilled the criteria of the International Study Group for Behcet's Disease, although some of the patients with RAU who did not fulfil the criteria presented with systemic manifestations such as those seen in Behcet's Disease (18.4% complained of neurologic symptoms, 10% presented with cutaneous manifestation, and 6% presented some articular manifestation) [44]. As such, diagnosis remains a challenge especially in low-prevalence countries such as Australia and New Zealand if based on the preexisting clinical criteria.

BD can cause significant morbidity and can be fatal. Immediate medical treatment is needed to prevent consequences. However, a number of patients do not fulfil ISG diagnostic criteria. This can lead to delay or misdiagnosis. Up to date, diagnosis depends mainly on a thorough medical history and meticulous clinical evaluation, keeping in mind that different manifestations can present in different subspecialties and can emerge at different points in time throughout the course of the disease. Increasing awareness among physicians can increase the diagnosis of Behcet's Disease and reduce morbidity.

## Figures and Tables

**Table 1:** Worldwide prevalence of Behcet's Disease [6].

Prevalence per 100,000	Country
421	Turkey
80	Iran
20	Saudi Arabia
17	Iraq
15.2	Israel
13.5	Japan
7.1	France
5.2	USA
4.9	Sweden
2.26	Germany
1.53	Portugal
0.64	UK

**Table 2:** Classification criteria for Behcet's Disease [45].

Classification criteria	Year
Curth	1946
Hewitt et al.	1969
Mason and Barnes	1969
Hewitt et al. revised	1971
Japan	1972
Hubault and Hamza	1974
O'Duffy	1974
Chen and Zhang	1980
Dilsen et al.	1986
Japan revised	1988
International Study Group	1990
Iran Traditional	1993
Iran Classification Tree	1993
Dilsen revised	2000
Korea	2003
International Criteria for Behcet's Disease	2006/2013 [17]

**Table 3:** The International Study Group criteria for Behcet's Disease [16].

Recurrent oral ulceration	Minor or major aphthous, or herpetiform ulceration observed by physician or patient, which recurred at least 3 times in one 12-month period
*Plus 2 of*	
Recurrent genital ulceration	Aphthous ulceration or scaring, observed by the physician or patient
Eye lesion	Anterior uveitis, posterior uveitis, or cells in vitreous on slit-lamp examination; or retinal vasculitis observed by an ophthalmologist
Skin lesions	Erythema nodosum observed by the physician or patient, pseudofolliculitis, or papulopustular lesions; acneiform nodules observed by the physician in postadolescent patients not on corticosteroid treatment
Positive pathergy test	Read by physician at 24–48 hours

(a) Findings applicable only in absence of other clinical explanations.

**Table 4:** International Criteria for Behcet's Disease [17].

Symptoms	Points
Oral aphthosis	2
Genital aphthosis	2
Ocular lesion	2
Skin lesion	1
Neurological manifestation	1
Vascular manifestation	1
Positive pathergy test	1^b^

(a) Point score system: scoring ≥4 indicates Behcet's diagnosis [17].

(b) Pathergy test is optional and the primary scoring system does not include pathergy testing. However, where pathergy testing is conducted, one extra point may be assigned for a positive result [17].

**Table 5:** Summary of recommended treatment [41, 42].

Manifestation	Treatment
*Mucocutaneous *	
Mild	Topical steroids
Moderate to severe	*Initial*:
	Systemic steroid
	Colchicine
	*Refractory case*:
	Azathioprine
	Interferon *α*
*Eye*	*Initial*:
	Systemic steroid + azathioprine
	*Refractory case*:
	(1st) Cyclosporine A + steroid + azathioprine
	(2nd) Interferon *α* +/− systemic steroid
*Arthritis*	(1st) Colchicine
	Nonsteroidal anti-inflammatory drug
	(2nd) Azathioprine
	Interferon *α*
*Vessels*	
Deep venous thrombosis	Azathioprine
	Cyclosporine A
	Cyclophosphamide (larger vessels)
Arterial aneurysm	Cyclophosphamide + systemic steroid
*Neurological *	Cyclophosphamide (drug of choice)
	Cyclosporine A (contraindicated)
*Gastrointestinal*	Systemic steroid
	Sulfasalazine
	Azathioprine
